# The Enhanced Lithium-Storage Performance for MnO Nanoparticles Anchored on Electrospun Nitrogen-Doped Carbon Fibers

**DOI:** 10.3390/nano8090733

**Published:** 2018-09-17

**Authors:** Rui Zhang, Xue Dong, Lechao Peng, Wenjun Kang, Haibo Li

**Affiliations:** 1 School of Chemistry and Chemical Engineering, Liaocheng University, Liaocheng 252059, China; ruizhangchn@sina.com (R.Z.); snowdong@163.com (X.D.); lechaopeng@163.com (L.P.); 2 Department of Chemical and Biomolecular Engineering, National University of Singapore, 10 Kent Ridge Crescent, Singapore 119260, Singapore

**Keywords:** manganese monoxide, carbon fibers, electrospinning, rate capability, cyclic stability, Li-ion battery

## Abstract

Manganese monoxide (MnO) is a promising anode material in the lithium-ion battery for its high capacity, low operation potential, and environmental benignity. However, its application is impeded by poor rate capability and rapid capacity fading. In this work, a MnO/carbon hybrid material, in which small-sized MnO nanoparticles are tightly anchored on carbon fibers (denoted as MnO@CFs), was prepared by annealing the electrospun precursor fibers at 650 °C. When applied as the anode material of the Li-ion battery, the small size of MnO shortens the Li-ion diffusion path, and the carbon fibers not only greatly improve the conductivity but also efficiently buffer the MnO structure strain during the charge–discharge process, endowing the MnO@CFs electrode with a good rate capability (185 mAh g^−1^ at 5 A g^−1^) and cyclic stability (406 mAh g^−1^ after 500 cycles at 1.0 A g^−1^).

## 1. Introduction

Graphite with a theoretical capacity of 372 mAh g^−1^ is a conventional anode material for the lithium-ion battery, however, the potential of Li-ion intercalation into graphite is close to 0 V, which causes the formation of Li dendrites and further induces short circuit of batteries [1]. Compared with graphite, MnO has a much higher theoretical capacity (756 mAh g^−1^), which can well meet the capacity requirement of the Li-ion battery [2]. However, its application is still impeded by poor rate capability and rapid capacity fading. The low rate capability originates from its low conductivity in essence, while the rapid capacity fading is usually caused by severe agglomeration and drastic volume change duringthe lithiation/delithiation process [3].

To address the above drawbacks, great efforts have been devoted to improving the MnO electrochemical performance. In the past 10 years, rational electrode design with nanomaterials has been proved to overcome well the problems associated with new battery chemistries [4]. For example, fabrication of the electrode with nanostructures such as nano/micro fibers and nanocomposites can resist mechanical degradation and large volume change during the electrochemical process. Nowadays, it is well known that coating or hybridizing MnO nanomaterials with carbon can efficiently enhance lithium storage performance, as it can efficiently improve the electrode contact interface and the lithium-ion diffusion path [5]. In previous studies, MnO/carbon hybrid materials, including graphene-loaded MnO [6,7,8,9], carbon nanofiber/nanotube-supported MnO [10,11], carbon-coated MnO nanoparticles/microspheres/nanowires [12,13,14,15,16], showed enhanced specific capacity, rate capability, and cyclic stability, when they were compared with pristine MnO counterpart. The introduction of a carbon component can highly improve the conductivity of MnO, what is more, the elastic feature of carbon materials can also relieve the strain caused by the volumetric change during the Li^+^ intersion/extraction process [17,18,19].

Recently, spinning techniques such as electrospinning, centrifugal jet spinning, and force-spinning have had a great application in the fabrication of metal oxide/carbon (MO_x_/C) composite materials, such as FeO_x_/C, CoO_x_/C, NiO/C, and SnO_2_/C, due to their versatility and facility [17,20,21,22,23,24,25,26]. The electrospinning technique is a simple and efficient strategy to prepare nano/micro fibers, which is applied in various fields, such as biomedicine and energy storage/conversion [27,28]. In order to obtain carbon fibers, polymers such as polyacrylonitrile (PAN), poly(vinyl alcohol) (PVA), poly(vinyl pyrrolidone) (PVP), poly(ethylene oxide) (PEO) are widely used as precursors [29], and the obtained electrospun carbon fibers not only suitably enhance electron transfer but also stabilize MO_x_ nanoparticles. Detailed electrochemical studies confirm that electrospun MO_x_/C hybrid materials exhibit an enhanced electrochemical Li-storage performance including rate capability and cyclic stability, when they are compared with the monomeric MO_x_ electrodes [30,31,32,33].

In this study, we applied an electrospinning technique to prepare a MnO/carbon hybrid material, in which small sized MnO nanoparticles (NPs) are tightly anchored on carbon fibers (denoted as MnO@CFs). The small size of MnO shortens the Li-ion diffusion path, and carbon fibers not only greatly improve the conductivity but also efficiently buffer MnO structure strain during the charge–discharge process. When applied as the anode materials of the Li-ion battery, the hybrid architecture endows the MnO@CFs electrode with a good rate capability and cyclic stability even at a high current density.

## 2. Materials and Methods 

### 2.1. Chemicals

Polyacrylonitrile (PAN) was supplied by Sigma-Aldrich Co. (St. Louis, MO, USA). Manganese(III) acetylacetonate (Mn(acac)_3_) was obtained from Alddin Industrial Co. (Shanghai, China). *N*,*N*-dimethylformamide (DMF) was purchased from Tanjin Fuyu Fine Chemical Co., Ltd. (Tianjin, China). 

### 2.2. Synthesis Procedure

To prepare MnO@CFs, 12.0 mL of DMF solution containing 0.79 g PAN and 1.69 g Mn(acac)_3_ was vigorously stirred for 10 h to form a transparent solution. Then it was loaded into a plastic syringe equipped with a 23 gauge spinning nozzle. The applied voltage was 10.8 kV, and the distance between nozzle and collector was 15.0 cm. The flow rate was controlled by a syringe pump at 6.0 μL min^–1^. The fibers were first stabilized at 230 °C for 3.0 h in air and then further annealed at 650 °C for 1.0 h under N_2_ atmosphere to obtain MnO@CFs. For comparison, carbon fibers (CFs) were also prepared by a similar electrospinning route in the absence of Mn(acac)_3_. Besides, MnO NPs were obtained by directly pyrolyzing Mn(acac)_3_ at 650 °C for 1.0 h under N_2_ atmosphere (Figure S1 in Supporting Information).

### 2.3. Characterization

Powder X-ray diffraction (XRD) was characterized by using a Philips X’pert X-ray diffractometer with Cu Kα radiation (λ = 1.5418 Å). The scanning electron microscopy (SEM) was performed on a Supra-40 scanning electron microscope (accelerating voltage: 5.0 kV, ZEISS, Oberkochen, Germany). For SEM characterization, the sample powder was directly fixed by conductive tape. The transmission electron microscopy (TEM) was performed on a JEM-2100 (accelerating voltage: 200 kV, JEOL, Tokyo, Japan). To characterize TEM, the sample was first dispersed in ethanol by ultrasonic treatment for 15 min, then transferred to a copper mesh by adding dropwise a suspension solution of the sample. Element distribution mapping characterization was carried out by an Oxford INCA energy dispersive X-ray detector equipped on JEM-2100. The X-ray photoelectron spectroscopy (XPS) study was accomplished by an ESCLAB MKII X-ray photoelectron spectrometer with a monochromatic Mg Kα X-ray source. The Raman spectrum was recorded on a Monovista-CRS500 confocal Raman spectrometer (Semiconductor Pacific Ltd. Hongkong, China). Thermogravimetric analysis (TGA) was performed on a DTG-60H analyzer (Shimadzu, Tokyo, Japan).

### 2.4. Electrochemical Measurements 

Electrochemical measurements were performed using CR2025 coin cells (Hefei Ke Jing Materials Technology Co., Ltd. Hefei, China). Active materials, carbon black, and poly(vinylidene fluoride) in *N*-methyl-2-pyrrolidone with weight ratio of 80:10:10 were mixed into a slurry. Then the slurry was pasted to a copper foil current collector (12 mm in diameter) at an active material loading of ~1.0 mg cm^−2^ (thickness ~6−8 µm), and vacuum-dried at 120 °C overnight. The mass capacities were calculated based on the composite. The electrolyte consisted of 1.0 M LiPF_6_ in ethylene carbonate/diethyl carbonate (1:1 by volume) solution. The galvanostatic charge–discharge test was performed using a LAND CT2001A system (Wuhan LAND electronics Co., Ltd., Wuhan, China) in the voltage range of 0.01–3.0 V. 

## 3. Results and Discussion

### 3.1. Structure and Morphology Characterization

**Figure 1:**
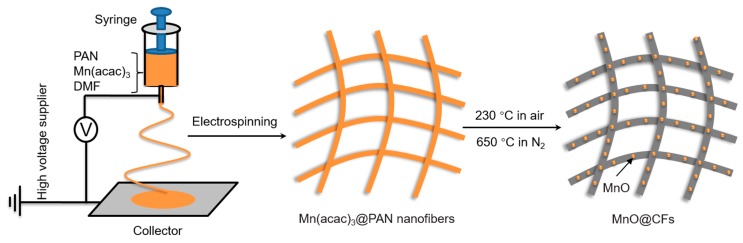
Schematic illustration of preparation of MnO nanoparticles anchored on carbon fibers (MnO@CFs).

**Figure 2:**
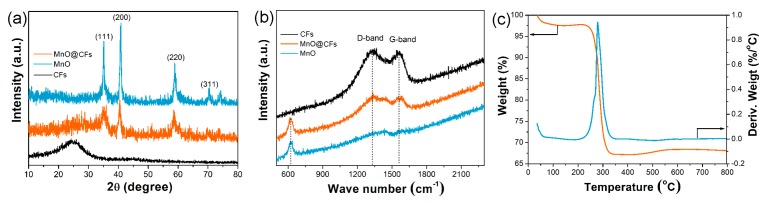
(**a**) Powder X-ray diffraction (XRD) patterns and (**b**) Raman spectra for MnO, CFs, and MnO@CFs. (**c**) Thermogravimetric analysis-differential thermogravimetry (TGA–DTG) curves for MnO@CFs in air atmosphere.

Figure 1 shows the schematic illustration for the synthesis of MnO@CFs, which involves two steps: electrospinning and annealing. For comparison, carbon fiber and pristine MnO counterparts were also prepared. Their phase compositions were investigated by powder XRD. When compared with the XRD patterns of CFs and MnO (Figure 2a), the peaks of MnO@CFs at 2*θ* = 35.0°, 40.6°, 58.8°, and 70.4° were well indexed to (111), (200), (220) and (311) reflections of cubic-phase MnO (JCPDS: No. 78-0424). Due to a low graphitization degree, the broad peak at 2*θ* = ~26°, corresponding to the (002) diffraction of carbon fibers (JCPDS: No. 41-1487), was not obvious for MnO@CFs. However, it could be further confirmed by Raman spectra. As shown in Figure 2b, MnO@CFs exhibit two strong peaks at 1332 and 1560 cm^−1^, respectively. The peak at 1560 cm^‒1^, named the G-band, corresponds to an E_2g_ vibration mode of sp^2^-bonded carbon atoms in graphene sheets. The peak at 1332 cm^−1^, named the D-band, is associated with carbon atoms with structural defects and disorder in the graphitic structure [34]. The intensity ratio of D and G bands (I_D_/I_G_) is calculated to be 1.49, also implying a low graphitization degree of carbon fibers. The low graphitization degree can be mainly attributed to the low carbonization temperature (650 °C), although the incorporation of nitrogen element in the carbon fibers also induces structure deformation. What is more, there is a Raman peak at 626 cm^−1^, which corresponds to the characteristic vibration of Mn–O in MnO [35]. We also applied the TGA technique to determine the MnO content in MnO@CFs (Figure 2c). The weight loss of 49.9 wt% originated from the integrative effects of the weight loss (H_2_O evaporation, carbon combustion) and the weight gain (MnO oxidation to Mn_2_O_3_) [36]. Based on the theoretical value (11.3 wt%) of weight increase from MnO to Mn_2_O_3_ [37], the MnO content was calculated to be 44.9 wt%.

**Figure 3:**
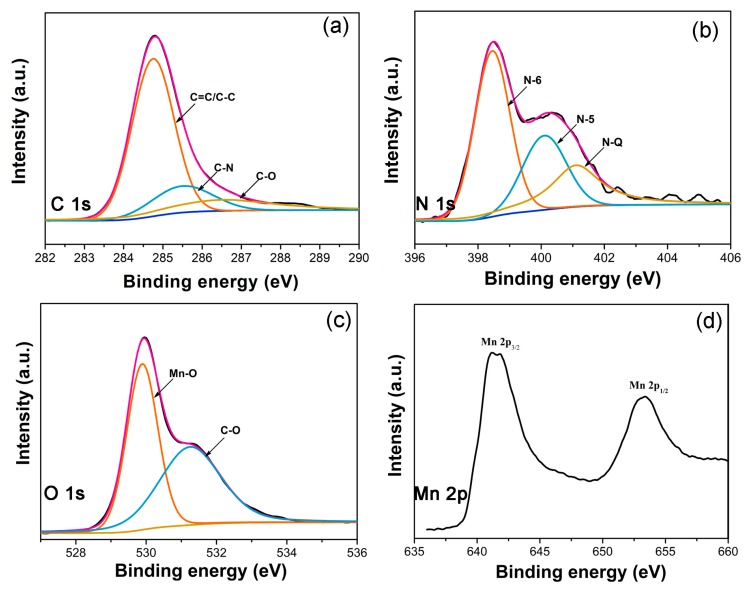
Peak-fitting X-ray photoelectron spectroscopy (XPS) spectra of (**a**) C1s (**b**) N1s (**c**) O1s and (**d**) Mn 2p for MnO@CFs.

XPS technique was used to study the surface element composition and electronic state of MnO@CFs. As shown in Figure S2, the survey spectrum confirms the presence of C, N, O, and Mn elements. It is noted that there are several chemical bond types for C, N, and O in MnO@CFs. The high resolution C1s XPS spectrum in Figure 3a can be fitted to three peaks (284.7, 285.5, and 286.5 eV), corresponding to C=C/C−C, C−N, and C−O, respectively [38]. On the basis of deconvolution of the N1s XPS spectrum (Figure 3b), there are also three types of nitrogen-containing species: pyridinic-type N (N-6, 398.5 eV), pyrrolic-type N (N-5, 400.1 eV), quaternary-type N (N-Q, 401.1 eV) [39]. Nitrogen-doping can improve the electrical conductivity of carbon fibers. The deconvolution of O1s XPS spectrum implies two kinds of oxygen-containing species: C−O (531.3 eV) and Mn−O (529.8 eV) (Figure 3c) [6,18]. It has been reported that the oxygen species in carbon materials can firmly bridge the carbon matrix and a MnO nanoparticle, which is beneficial to stabilizing the MnO nanoparticle [40]. What is more, there are two characteristic peaks for Mn 2p, corresponding to Mn 2p_3/2_ (641.4 eV) and Mn 2p_1/2_ (653.3 eV), respectively, and the result is very consistent with previous reports [5,20].

**Figure 4:**
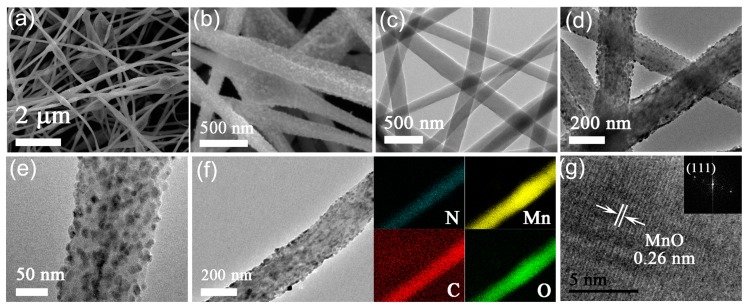
(**a**,**b**) Scanning electron microscopy (SEM) and (**d**,**e**) transmission electron microscopy (TEM) images of MnO@CFs. (**c**) TEM image of CFs. (**f**) Element mapping of C, N, O, and Mn for an individual MnO@CFs. (**g**) high-resolution TEM (HRTEM) image of a random MnO nanoparticle on CFs.

TEM and SEM were applied to investigate the microstructures of products. The SEM images in Figure 4a,b reveal that MnO@CFs have a fibrous structure. Compared with pure CFs (Figure 4c), MnO@CFs show an obviously rough surface (Figure 4d). TEM characterization confirms that small MnO nanoparticles with sizes of ~10–15 nm are uniformly anchored on the surfaces of the carbon fibers (Figure 4e). The carbon fibers exhibit a strong fixation for MnO nanoparticles, and there is no obvious MnO abscission even after a long sonication treatment. We believe that the strong interaction can effectively fix the MnO nanoparticle and also facilitate electron transfer during the electrochemical process. The element mapping in Figure 4f shows a good distribution for C, N, Mn, and O across the randomly selected MnO@CFs. To further confirm the phase of anchored nanoparticles, high-resolution TEM (HRTEM) technique was used to study the lattice spacing of a random nanoparticle. The HRTEM image in Figure 4g shows a well-defined lattice spacing of 0.26 nm, which is very consistent with cubic-phase MnO (111) crystalline plane. It verifies very well the MnO phase of nanoparticles on carbon fibers.

### 3.2. Electrochemical Performances

**Figure 5:**
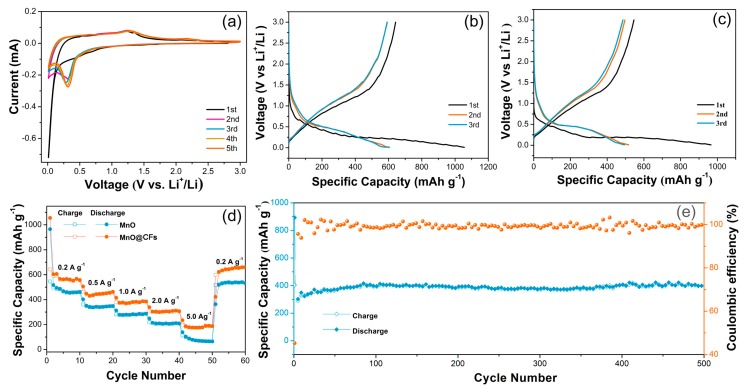
(**a**) Cyclic voltammetry (CV) of MnO@CFs electrode. Galvanostatic charge–discharge curves of (**b**) MnO@CFs and (**c**) MnO electrodes at 0.2 A g^−1^. (**d**) Rate performance of MnO@CFs and MnO electrodes with different current densities. (**e**) Long-term cyclic performance and coulombic efficiency of the MnO@CFs electrode at 1.0 A g^−1^.

We first investigated the electrochemical performance by cyclic voltammetry (CV). As shown in Figure 5a, the cathodic peak in the first sweep starts from ~0.82 V due to the formation of a solid-electrolyte interface (SEI) layer, which is inevitable on the surface of electrodes. The sharp reduction peak close to 0.01 V is ascribed to the reduction of Mn^2+^ to Mn^0^ (MnO + 2Li^+^ + 2e^‒^ → Mn + Li_2_O) [35]. This peak shifts to ~0.31 V in the following cycles, which is attributed to the accelerated kinetics and microstructure change of the MnO@CFs electrode after the first lithiation [13]. The anodic peak at ~1.25 V corresponds to the oxidation of Mn^0^ to Mn^2+^ and Li_2_O decomposition (Mn + Li_2_O →MnO + 2Li^+^ + 2e^‒^) [35,40]. It is of note that the CV profiles basically overlap after two cycles, demonstrating a good reversibility of the electrochemical reaction.

Figure 5b shows the charge–discharge profiles of MnO@CFs for the initial three cycles at 0.2 A g^−1^. Two voltage plateaus at ~0.5 and ~1.2 V during the discharge/charge process are very consistent with the CV curves. The first discharge/charge capacities are 1050 and 640 mAh g^−1^, respectively, and the initial coulombic efficiency is 61%, being higher than the 56% of pristine MnO (Figure 5c). It is worth noting that the high coulombic efficiency of the first cycle is of benefit for the full cell performance, as the low coulombic efficiency in the first cycle means extra Li^+^ supply from the cathode part. The capacity loss in the first cycle mainly comes from the irreversible processes including electrolyte decomposition and SEI layer formation, which is common to most oxide anode materials and has also been observed in the previous literature on MnO electrodes [40,41,42]. The rate capability of MnO@CFs was evaluated at different current densities from 0.2 to 5.0 A g^−1^ (Figure 5d). The stable capacities of MnO@CFs electrode at 0.2, 0.5, 1.0, and 2.0 A g^−1^ reach 570, 450, 385, and 305 mAh g^−1^, which are higher than 460, 345, 285, and 205 mAh g^−1^ of the MnO electrode at the same current density. When the current densities are raised from 0.2 to 2.0 A g^−1^, the capacity decrease for MnO is 55%, but the value for MnO@CFs is much less (46%). What is more, MnO@CFs still delivers a discharge capacity of 185 mAh g^−1^ at 5.0 A g^−1^, however, the value for MnO dramatically declines to 60 mAh g^−1^. It can be concluded that MnO@CFs exhibit a better rate capability than MnO, especially at high current density, and it also confirms the key role of carbon fibers in improving the rate capability of MnO. 

To evaluate the long-term cyclic stability, the MnO@CFs electrode is galvanostatically charged and discharged at 1.0 A g^−1^ for 500 cycles (Figure 5e). The initial reversible capacity is 377 mAh g^−1^. Its discharge capacity sluggishly declines during the first 10 cycles, then it slightly rises and keeps a capacity value of 406 mAh g^−1^ after 500 cycles. Moreover, its coulombic efficiency nearly remains 100% throughout the overall cycling range except for the 1st cycle. The increase of capacity of the first 100 cycles is due to the reversible growth of a polymeric gel-like film, which results from kinetically activated electrolyte degradation [43]. For the MnO electrode, it shows a gradual decline trend after ~200 cycles and reaches a discharge capacity of 323 mAh g^−1^ at the 500th cycle (Figure S3). The good rate performance and cyclic stability are attributed to the unique architecture of MnO@CFs, as the small size of MnO can highly shorten the Li-ion diffusion distance, and carbon fibers can serve as a good conductive matrix and also buffer the structure strain derived from the MnO volume change. We also investigated the structure integrity of MnO@CFs after 500 charge–discharge cycles at a current density of 1.0 A g^−1^. As shown in Figure S4, MnO nanoparticles are well anchored on carbon fiber surfaces even after repeated lithiation/delithiation processes, and there is no obvious MnO stripping, implying a good structure stability of MnO@CFs. 

**Figure 6:**
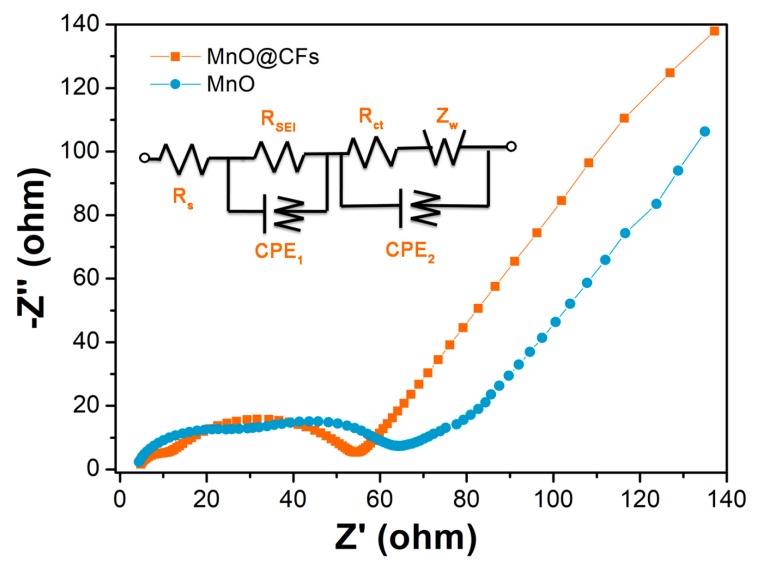
Nyquist plots for the MnO@CFs and MnO electrodes. Inset shows the equivalent circuit.

Electrochemical impedance spectra (EIS) is a powerful technique to analyze the electrode kinetics. The EIS for MnO@CFs and MnO electrode was conducted at 1.2 V after five galvanostatic discharge–charge cycles, and the impedance patterns were recorded in the frequency range from 10 mHz to 100 kHz. As shown in Figure 6, each Nyquist plot consists of a depressed semicircle at high frequency and a linear Warburg part at low frequency, which are associated with the charge–transfer reaction process at the electrode/electrolyte and Li-ion diffusion in the bulk electrode, respectively [6,19]. To betterl depict the EIS result, the equivalent circuit is also provided (inset of Figure 6). The R_s_, R_SEI_, and R_ct_ represent the electrolyte resistance, SEI film resistance, and charge transfer resistance, respectively. CPE_1_ and CPE_2_ are the corresponding constant phase elements about the double layer capacitance, and Z_w_ is the Warburg impedance. On the basis of the equivalent circuit, the fitting R_s_, R_SEI_, and R_ct_ for MnO@CFs are 4.4, 5.9, and 44 Ω, and the ones for MnO are 3.8, 14.2, and 51 Ω. It can be confirmed that the overall total impedance value for MnO@CFs is lower than that of MnO. As it involves multiple electron/ion transfer during the discharge–charge process, so the lower resistance implies a good electrode kinetics, and it correlates well with the galvanostatic discharge–charge performance. In the case of MnO@CFs, CFs contact well with MnO nanoparticles, which not only greatly improves the electronic conductivity but also significantly reduces the charger transfer resistance. 

## 4. Conclusions

In summary, MnO@CFs were prepared by a convenient electrospinning approach, and their lithium-storage performance investigated. Due to the shortened Li-ion diffusion pathway, enhanced electronic conductivity and improved strain buffer for MnO volume change, the unique architecture endows MnO@CFs with a good rate capability and cyclic stability, making it an alternative anode material for the Li-ion battery.

## Supplementary Materials

The following are available online at http://www.mdpi.com/2079-4991/8/9/733/s1, Figure S1: TEM images of MnO NPs obtained by directly pyrolyzing Mn(acac)_3_ at 650 °C for 1.0 h under N_2_ atmosphere, Figure S2: XPS survey spectrum of MnO@CFs, Figure S3: Long-term cyclic performance and coulombic efficiency of MnO electrode at a current density of 1.0 A g^‒1^, Figure S4: TEM and HRTEM images of MnO@CFs after 500 charge-discharge cycles at a current density of 1.0 A g^‒1^.
