# Supplementary material for: The Enhanced Lithium-Storage Performance for MnO Nanoparticles Anchored on Electrospun Nitrogen-Doped Carbon Fibers
Source: Nanomaterials (Basel). 2018 Sep 17;8(9):733. doi: 10.3390/nano8090733 (PMC6163262; doi:10.3390/nano8090733)
Supplement: Supplementary file 1 [file nanomaterials-08-00733-s001.zip › nanomaterials-359337-supplementary.docx]

**Supporting Information**

The Enhanced Lithium-Storage Performance for MnO Nanoparticles Anchored on Electrospun Nitrogen-Doped Carbon Fibers

Rui Zhang ^1^, Xue Dong ^1^, Lechao Peng ^1^, Wenjun Kang ^1,^* and Haibo Li ^1,2,^*

^1^ School of Chemistry and Chemical Engineering, Liaocheng University, Liaocheng 252059, China; ruizhangchn@sina.com (R.Z.); snowdong@163.com (X.D.); lechaopeng@163.com (L.P)

^2^ Department of Chemical and Biomolecular Engineering, National University of Singapore, 10 Kent Ridge Crescent, Singapore 119260, Singapore

***** Correspondence: kangwenjun@lcu.edu.cn (W.K.); haiboli@mail.ustc.edu.cn or chev175@edu.nus.sg (H.L.)


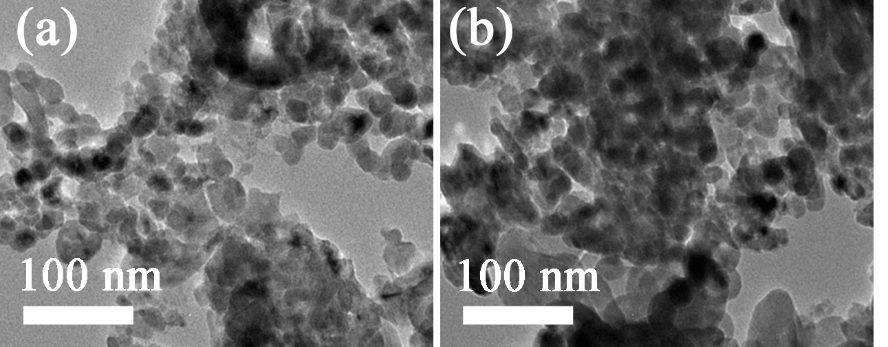


**Figure S1.** TEM images of MnO nanoparticles obtained by directly pyrolyzing Mn(acac)_3_ at 650 °C for 1.0 h under N_2_ atmosphere.


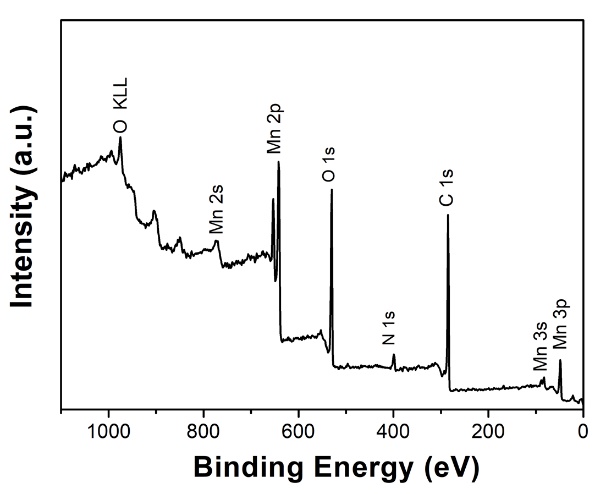


**Figure S2.** X-ray photoelectron spectroscopy (XPS) survey spectrum of MnO nanoparticles anchored on carbon fibers (MnO@CFs).


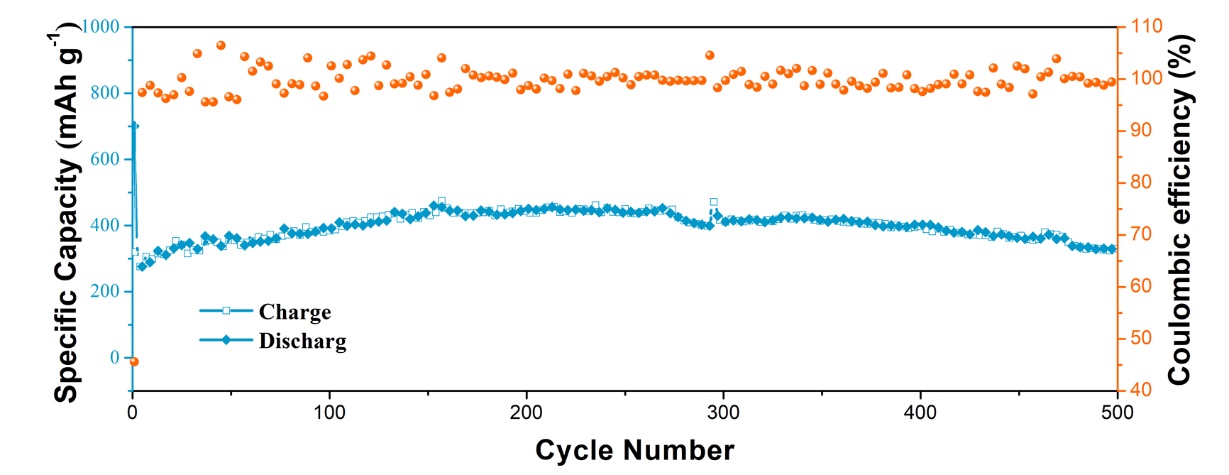


**Figure. S3**. Long-term cyclic performance and Coulombic efficiency of MnO electrode at a current density of 1.0 A g^−1^.


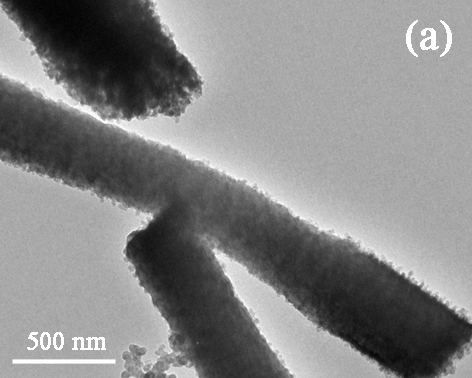

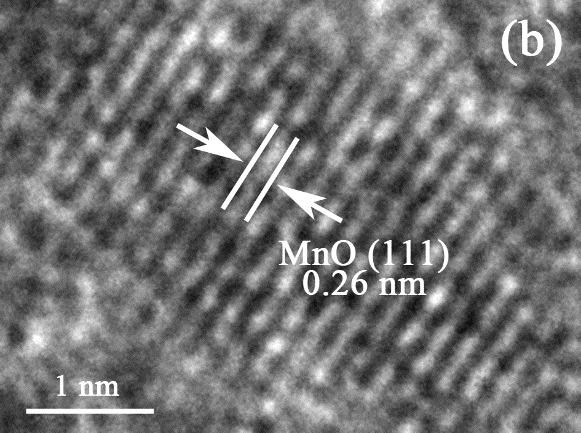


**Figure S4.** (**a**) Transmission electron microscopy (TEM) and (**b**) high-resolution TEM (HRTEM) images of MnO@CFs after 500 charge-discharge cycles at a current density of 1.0 A g^−1^.
